# Supplementary material for: Inhibitory Effects of IL-6-Mediated Matrix Metalloproteinase-3 and -13 by Achyranthes japonica Nakai Root in Osteoarthritis and Rheumatoid Arthritis Mice Models
Source: Pharmaceuticals (Basel). 2021 Aug 7;14(8):776. doi: 10.3390/ph14080776 (PMC8402178; doi:10.3390/ph14080776)
Supplement: Supplementary file 1 [file pharmaceuticals-14-00776-s001.zip › pharmaceuticals-1292253-supplementary.pdf]

Inhibitory effects of IL-6-mediated matrix metalloproteinase-3 and -13 by *Achyranthes japonica* Nakai root in osteoarthritis and rheumatoid arthritis mice models

Xiangyu Zhao, Dahye Kim, Jiwon Yang, Mangeun Kim, Yaping Zhao, Mrinmoy Ghosh, Young-Ok Son

Supplementary Information

Supplementary Table S1. PCR primers and conditions

Genes	Strand	Primer Sequences	Size (bp)	AT (℃)	Origin
Gapdh	A<br>AS	5′-TCACTGCCACCCAGAAGAC-3′<br>5′-TGTAGGCCATGAGGTCCAC-3′	450	57.3	Mouse
Mmp2	A<br>AS	5′-CCAACTACGATGATGAC-3′<br>5′-ACCAGTGTCAGTATCAG-3′	233	60.0	Mouse
Mmp3	A<br>AS	5′-AGGGATGATGATGCTGGTATGG-3′<br>5′-CCATGTTCTCCAACTGCAAAGG-3′	434	58.0	Mouse
Mmp9	A<br>AS	5′-TGCACTGGGCTTAGATCATTCC-3′<br>5′-CCGTCCTTGAAGAAATGCAGAG-3′	450	58.4	Mouse
Mmp10	A<br>AS	5′-AGAAATGGACACTTGCACCCTCAG-3′<br>5′-CTGTCCGTGTTGTGAGCCTCATAG-3′	448	61.5	Mouse
Mmp12	A<br>AS	5′-CCCAGAGGTCAAGATGGATG-3′<br>5′-GGCTCCATAGAGGGACTGAA-3′	482	60.0	Mouse
Mmp13	A<br>AS	5′-TGATGGACCTTCTGGTCTTCTGG-3′<br>5′-CATCCACATGGTTGGGAAGTTCT-3′	474	58.2	Mouse
Mmp14	A<br>AS	5′-GTGCCCTATGCCTACATCCG-3′<br>5′-TTGGGTATCCATCCATCACT-3′	580	58.0	Mouse
Mmp15	A<br>AS	5′-GAGAGATGTTTGTGTTCAAGGG-3′<br>5′-TGTGTCAATGCGGTCATAGGG-3′	260	57.5	Mouse
Adamts 4	A<br>AS	5′-CATCCGAAACCCTGTCAACTT-3′<br>5′-GCCCATCATCTTCCACAATAGC-3′	287	58.4	Mouse
Adamts 5	A<br>AS	5′-GCCATTGTAATAACCCTGCACC-3′<br>5′-TCAGTCCCATCCGTAACCTTTG-3′	292	58.4	Mouse
Aggrecan	A<br>AS	5’-GAAGACGACATCACCATCCAG-3’<br>5’CTGTCTTTGTCACCCACACAT-3’	581	60.0	Mouse
Col2a1	A<br>AS	5’-CACACTGGTAAGTGGGGCAAGACCG-3’<br>5’-GGATTGTGTTGTTTCAGGGTTCGGG-3’	173	58.0	Mouse
Epas1	A<br>AS	5’-CGAGAAGAACGACGTGGTGTTC-3’<br>5’-GTGAAGGCTGGCAGGCTCC-3’	333	64.0	Mouse
Sox9	A<br>AS	5’-ATGCTATCTTCAAGGCGCTG-3’<br>5’-GACGTCGAAGGTCTCAATGT-3’	272	60.0	Mouse
Slc39a8	A<br>AS	5’-GAACAATTGCCTGGATGATCACGC-3’<br>5’-AAGCCGGTTAACATCCCTGCATTC-3’	430	63.0	Mouse

AT, annealing temperature; S, sense; AS, antisense

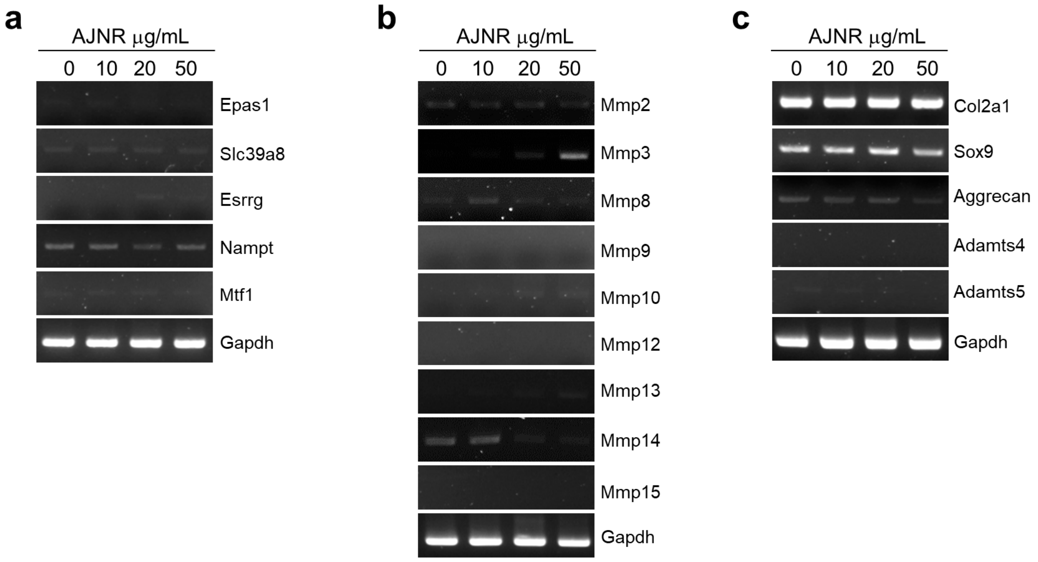

Supplementary Figure S1. Effects of AJNR on anabolic or catabolic factors in primary cultured articular chondrocytes. Primary cultured mouse chondrocytes were exposed to AJNR (0-50 μg/mL) for 24 h. Next, the levels of osteoarthritis inducer genes (A), matrix metalloproteinases (B), and anabolic/catabolic factors (C) were measured by RT-PCR. The results are representative of three independent experiments from different mouse pups. Glyceraldehyde-3-phosphate dehydrogenase (Gapdh) was used as an internal marker. Epas1, endothelial PAS domain protein 1; Slc39a8, solute carrier family 39 member 8; Esrrg, estrogen-related receptor gamma; Nampt, nicotinamide phosphoribosyltransferase; Mtf1, metal regulatory transcription factor 1; Mmp2, -3, -8, -9, -10, -12, -13, -14, and -15, matrix metalloproteinase-2, -3, -8, -9, -10, -12, -13, -14, and -15; Col2a1, collagen type II alpha 1 chain; Sox9, SRY-Box transcription factor 9; Adamts-4 and -5, ADAM metallopeptidase with thrombospondin type 1 motif-4 and -5
